# Mortality trends of cardiac, mediastinal and pleural malignancies in the United States, 1999–2020: a population-based analysis of demographic and geographic disparities

**DOI:** 10.3389/fonc.2026.1796015

**Published:** 2026-05-08

**Authors:** Wenlong Ding, Fachao Shi, Siyun Li, Siwen Xue, Cunming Fang, Caoyang Fang

**Affiliations:** 1 Department of Cardiology,Xuancheng Hospital Affiliated to Wannan Medical College (Xuancheng People ‘s Hospital), Xuancheng, Anhui, China; 2 Department of Cardiology,Maanshan People’s Hospital,Maanshan Hospital Affiliated to Wannan Medical College, Maanshan, Anhui, China; 3 Department of Emergency,The First Affiliated Hospital of USTC,Division of Life Sciences and Medicine, University of Science and Technology of China, Hefei, Anhui, China

**Keywords:** cardiac neoplasms, health disparities, joinpoint regression, mediastinal neoplasms, mortality trends, pleural neoplasms

## Abstract

**Background:**

Cardiac, mediastinal, and pleural malignancies represent rare but clinically significant thoracic neoplasms. Despite their clinical importance, comprehensive epidemiological analyses of mortality trends for these tumors remain limited. This study aimed to analyze mortality trends and demographic disparities of these malignancies in the United States from 1999 to 2020.

**Methods:**

We conducted a retrospective observational study using the Centers for Disease Control and Prevention’s Wide-ranging Online Data for Epidemiologic Research (CDC WONDER) database. Adults (≥18 years) who died from cardiac, mediastinal, or pleural malignancies (ICD-10 code: C38) between January 1, 1999, and December 31, 2020, were included. Age-adjusted mortality rates (AAMRs) were calculated and stratified by sex, age, race/ethnicity, geographic region, and urbanization level. Joinpoint regression analysis was employed to identify temporal trends, with calculation of annual percent changes (APCs) and average annual percent changes (AAPCs).

**Results:**

During the study period, 5,424 deaths were attributed to cardiac, mediastinal, or pleural malignancies, with an overall AAMR of 0.109 per 100,000 population (95% CI: 0.105-0.112). Males demonstrated higher mortality rates than females (0.133 vs. 0.076 per 100,000). Elderly individuals (≥65 years) accounted for 65.08% of total deaths. Overall mortality exhibited a significant declining trend (AAPC: -4.325%; 95% CI: -4.890 to -3.191; P<0.001), characterized by rapid initial decline from 1999-2001 (APC: -15.245%; P<0.001), followed by a more gradual decrease from 2001-2020 (APC: -3.096%; P = 0.044). Significant disparities were observed across demographic and geographic variables. White individuals had the highest AAMR (0.109 per 100,000), followed by African Americans (0.097) and Hispanic or Latino populations (0.059). Geographic analysis revealed the highest mortality rates in the Western region (0.131 per 100,000) and lowest in the Midwest (0.085). Metropolitan areas demonstrated more pronounced mortality decline (AAPC: -3.922%) compared to non-metropolitan areas (AAPC: -2.309%). State-level analysis revealed substantial heterogeneity, with Washington State exhibiting the highest AAMR (0.172 per 100,000) and Connecticut the lowest (0.056).

**Conclusions:**

Over the past two decades, mortality from cardiac, mediastinal, and pleural malignancies in the United States has significantly declined; however, substantial demographic and geographic disparities persist. These findings underscore the need for targeted interventions to address regional disparities and ensure equitable access to early detection and treatment strategies across all populations.

## 1 Introduction

Cardiac, mediastinal, and pleural malignancies constitute a group of rare but clinically significant thoracic neoplasms characterized by low incidence but often poor prognosis (1). While primary lesions of these tumors are relatively uncommon, accounting for approximately 0.1-0.3% of all malignancies, metastatic disease is more frequently encountered (2). Primary cardiac malignancies have an annual incidence rate of 0.0017-0.056%, with sarcomas being the most common type, comprising over 75% of cases (3). Mediastinal malignancies encompass thymomas, germ cell tumors, and neurogenic tumors, representing 3-7% of all thoracic neoplasms (4). Malignant pleural mesothelioma, the most common primary pleural malignancy, is closely associated with asbestos exposure and demonstrates increasing global incidence rates (5).

In recent years, advances in imaging technology and the development of multidisciplinary treatment approaches have improved early detection rates for these malignancies (6). The widespread implementation of computed tomography (CT), magnetic resonance imaging (MRI), and positron emission tomography-computed tomography (PET-CT) has enabled precise tumor localization and staging (7). Simultaneously, surgical technique improvements, including minimally invasive and robot-assisted procedures, have significantly enhanced patient outcomes (8). The emergence of targeted therapies and immunotherapy has provided novel treatment options for advanced-stage patients, with notable survival prolongation observed in selected cases (9, 10).

However, the epidemiological characteristics and mortality trends of these malignancies demonstrate significant variations across different populations. Previous studies have indicated that factors including age, sex, race, and geographic location can influence both tumor incidence and prognosis (11). Analysis of the United States Surveillance, Epidemiology, and End Results (SEER) database revealed that cardiac sarcomas have a median survival of only 11 months, with 5-year survival rates below 20% (12). Large-scale European cohort studies have identified more than 10-fold differences in mesothelioma incidence and mortality rates between countries, correlating closely with industrialization levels and historical asbestos usage patterns (13). Asian studies have demonstrated distinct pathological distributions of mediastinal tumors compared to Western countries, with higher proportions of thymomas and germ cell tumors (14).

Socioeconomic factors play a crucial role in the diagnosis and treatment of these malignancies. Research has shown that patients with lower socioeconomic status tend to present with later-stage disease, receive standard treatment less frequently, and experience poorer survival outcomes (15). Healthcare insurance coverage, unequal distribution of medical resources, and geographic accessibility all potentially influence patient treatment outcomes (16). Urban-rural disparities are particularly pronounced in cancer mortality rates, with rural patients facing challenges including diagnostic delays, specialist shortages, and limited access to advanced therapeutic technologies (17).

Despite individual studies reporting epidemiological characteristics of specific tumor types, systematic analyses of long-term mortality trends for cardiac, mediastinal, and pleural malignancies collectively remain lacking (18). Previous research has predominantly relied on single-center or regional data with limited sample sizes, failing to reflect the true national landscape (19). Furthermore, most studies have inadequately considered the impact of demographic and geographic factors on mortality trends and lacked precise identification of temporal trend inflection points (20). Understanding the spatiotemporal distribution characteristics of mortality from these tumors and their influencing factors is critically important for developing targeted prevention strategies, optimizing healthcare resource allocation, and improving patient outcomes (21).

Therefore, this study utilized national mortality registry data from the Centers for Disease Control and Prevention to systematically analyze mortality trends of cardiac, mediastinal, and pleural malignancies from 1999 to 2020, while examining the impact of sex, age, race, geographic region, and urbanization on mortality rates. The findings aim to provide scientific evidence for public health policy development and clinical practice improvement.

## 2 Materials and methods

### 2.1 Study design and data sources

This study employed a retrospective observational design with data sourced from the Wide-ranging Online Data for Epidemiologic Research (CDC WONDER) database of the Centers for Disease Control and Prevention (CDC), specifically utilizing the Multiple Cause of Death (MCOD) files. CDC WONDER encompasses death certificate data from all 50 U.S. states and the District of Columbia, maintained centrally by the National Center for Health Statistics (NCHS). Each mortality record contains demographic information and both underlying and contributing causes of death, coded according to the International Classification of Diseases, Tenth Revision (ICD-10), ensuring comprehensive national coverage and coding consistency. All data were extracted online through the official CDC WONDER website (https://wonder.cdc.gov).

### 2.2 Study subjects

The study population comprised adults (≥18 years) registered as deaths within the United States between January 1, 1999, and December 31, 2020, with cardiac, mediastinal, or pleural malignancies listed as the underlying cause of death. Cardiac, mediastinal, or pleural malignancies were defined according to ICD-10 code C38, which includes: malignant neoplasm of heart (C38.0), malignant neoplasm of anterior mediastinum (C38.1), malignant neoplasm of posterior mediastinum (C38.2), malignant neoplasm of mediastinum, part unspecified (C38.3), malignant neoplasm of pleura (C38.4), and malignant neoplasm of overlapping sites of heart, mediastinum and pleura (C38.8).It should be noted that the mediastinal tumor spectrum also encompasses lymphomatous malignancies, including Hodgkin’s lymphoma and non-Hodgkin’s lymphoma particularly primary mediastinal large B-cell lymphoma,which represent some of the most frequently encountered mediastinal malignancies in clinical practice, especially among younger patients. These entities, classified under ICD-10 codes C81–C86, are not captured within the C38 coding framework and therefore fall outside the scope of the present analysis.

### 2.3 Definition of variables

The analytical framework of this study encompassed categorical variables including age, sex, race/ethnicity, urbanization level, state, and census region. Sex was classified using a binary categorization of male and female. Racial and ethnic composition included Hispanic or Latino, Black or African American, and White populations. Based on the 2013 U.S. Census standards, residential locations were categorized by urbanization level into urban areas (encompassing large metropolitan areas and small-to-medium metropolitan areas) and rural areas (counties with populations below 50,000). Geographic regions followed the U.S. Census Bureau classification system, comprising four major regions: Northeast, Midwest, South, and West.

### 2.4 Statistical analysis

Trend assessment of mortality rates from 1999 to 2020 was conducted using the Joinpoint Regression Program (version 4.9.1) recommended by the National Cancer Institute. The model enables detection of inflection points (joinpoints) within time series data. For each analysis, we calculated the annual percent change (APC) and corresponding 95% confidence intervals (95% CI). The APC represents the annual relative percentage change in mortality rates during specific time periods. Additionally, we computed the average annual percent change (AAPC) for the entire study period (1999-2020), which represents a weighted average of multiple APC values. All statistical tests were two-sided, with P<0.05 considered statistically significant. Statistical analyses other than joinpoint regression were performed using R software (version 4.3.0).

## 3 Results

### 3.1 Overall mortality characteristics and trends

From 1999 to 2020, 5,424 individuals died from cardiac, mediastinal, or pleural malignancies in the United States. The overall age-adjusted mortality rate (AAMR) was 0.109 per 100,000 population (95% CI: 0.105-0.112) (**Table 1**). Of these deaths, 2,931 (54.04%) occurred in males and 2,493 (45.96%) in females. By race/ethnicity, deaths included 168 Hispanic or Latino individuals, 544 Black or African American individuals, and 4,712 White individuals. Age distribution analysis revealed 540 deaths (9.96%) among young adults aged 25-44 years, 1,354 deaths (24.96%) among middle-aged adults aged 45-64 years, and 3,530 deaths (65.08%) among elderly individuals aged ≥65 years (**Table 1**; **Supplementary Table S1**).

**Table 1:** Demographic characteristics and age-adjusted mortality rates (AAMR) for cardiac, mediastinal, and pleural malignancies in the United States, 1999–2020.

Variables	Deaths	Population	AAMR (95%CI)
Overall	5424	4473854489	0.109 (0.105-0.112)
Sex			
Male	2931	2154556911	0.133 (0.128-0.138)
Female	2493	2319297578	0.076 (0.073-0.079)
Race/Ethnicity			
Hispanic or Latino	168	295429555	0.059 (0.048-0.070)
Black or African American	544	546447527	0.097 (0.088-0.106)
White People	4712	3631977407	0.109 (0.105-0.112)
Census Region			
Northeast	824	827193779	0.097 (0.090-0.104)
Midwest	1166	969567311	0.085 (0.080-0.091)
South	2052	1652256217	0.109 (0.103-0.114)
West	1382	1024837182	0.131 (0.123-0.138)
Urbaniztion			
Metropolitan (Urban)	4377	3795213822	0.109 (0.105-0.112)
Nonmetropolitan (Rural)	1047	678634169	0.119 (0.111-0.127)
Ten-Year Age Groups			
25-44 years	540	1851376757	0.029 (0.027-0.032)
45-64 years	1354	1694001067	0.080 (0.076-0.084)
65+ years	3530	928476665	0.380 (0.367-0.393)

AAMR, age‐adjusted mortality rate; CI, confidence interval.

**Figure 1:**
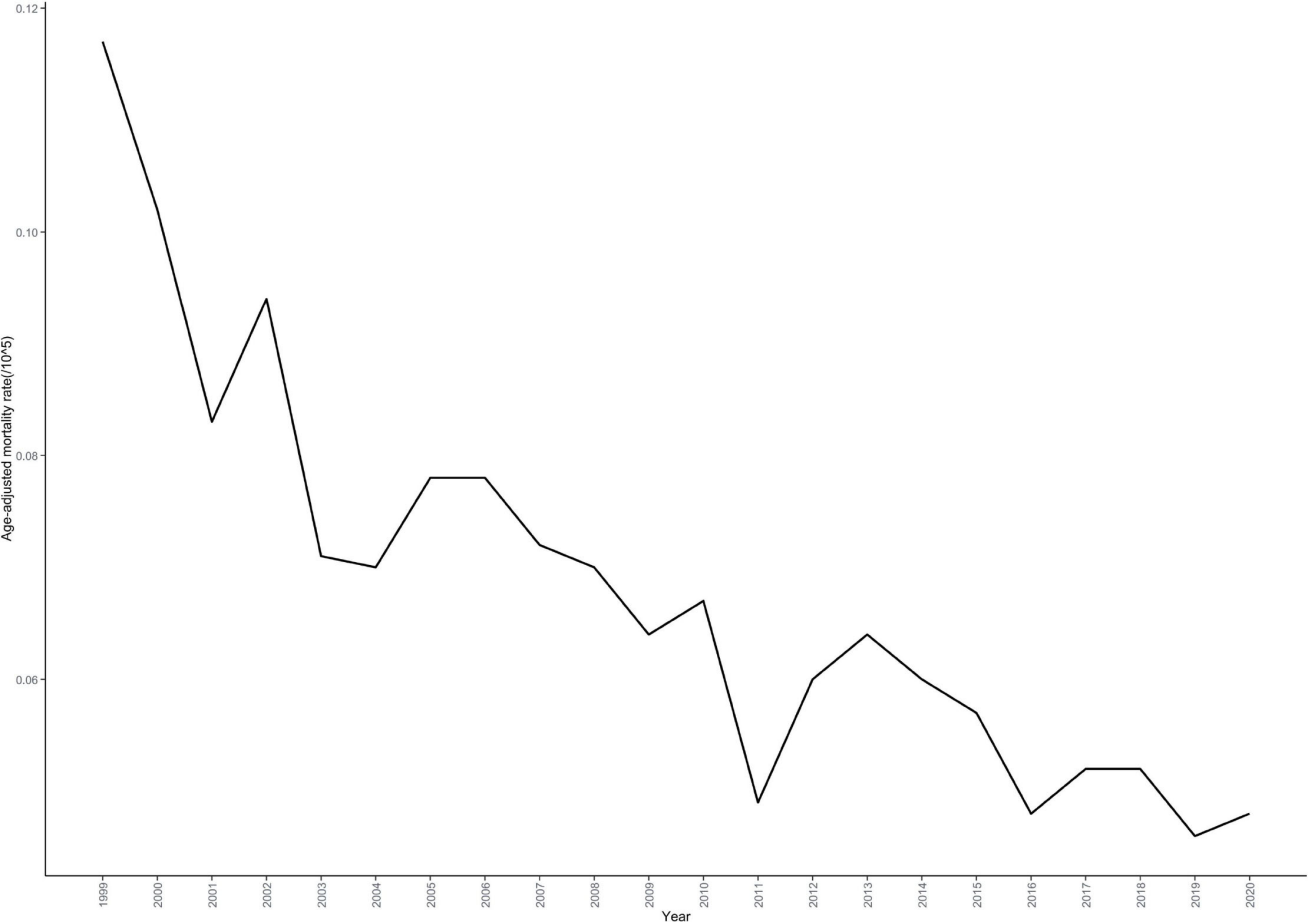
Overall age-adjusted mortality rate trends for cardiac, mediastinal, and pleural malignancies in the United States, 1999–2020.

Joinpoint regression analysis demonstrated a declining trend in mortality rates associated with cardiac, mediastinal, or pleural malignancies. The average annual percent change (AAPC) was -4.325% (95% CI: -4.890 to -3.191; P < 0.001). Two distinct trend segments were identified: an initial sharp decline from 1999 to 2001 (APC: -15.245%; 95% CI: -20.538 to -3.731; P < 0.001), followed by an attenuated declining trend from 2001 to 2020 (APC: -3.096%; 95% CI: -3.807 to -0.397; P = 0.044) (**Table 2**; **Figure 1**).

**Table 2:** Joinpoint regression analysis of mortality trends for cardiac, mediastinal, and pleural malignancies by demographic characteristics in the United States, 1999–2020.

Variables	Trend segment	Year interval	APC (95% CI)	AAPC (95% CI)	*P-*value
Entire Cohort	–	1999-2020	–	-4.325 (-4.890 to -3.191)	<0.001
	1	1999-2001	-15.245 (-20.538 to -3.731)	–	<0.001
	2	2001-2020	-3.096 (-3.807 to -0.397)	–	0.044
Sex					
Male	–	1999-2020	–	-3.233 (-4.273 to -2.211)	<0.001
Female	–	1999-2020	–	-3.133 (-4.296 to -1.980)	<0.001
Age					
25-44	–	1999-2020	–	-3.586 (-4.519 to -2.258)	<0.001
	1	1999-2004	-9.226 (-20.644 to -3.313)	–	0.015
	2	2004-2020	-1.753 (-3.100 to 4.573)	–	0.288
45-64	–	1999-2020	–	-3.200 (-3.820 to -2.566)	<0.001
65+	–	1999-2020	–	-2.503 (-3.377 to -1.627)	<0.001
Race/Ethnicity					
White People	–	1999-2020	–	-3.153 (-3.808 to -2.471)	<0.001
Census Region					
Northeast	–	1999-2020	–	-2.310 (-3.606 to -1.011)	0.001
Midwest	–	1999-2020	–	-3.769 (-4.967 to -2.591)	<0.001
South	–	1999-2020	–	-2.778 (-3.877 to -1.623)	<0.001
West	–	1999-2020	–	-3.517 (-5.331 to -1.642)	0.001
Urbaniztion					
Metropolitan (Urban)	–	1999-2020	–	-3.922 (-4.731 to -2.760)	<0.001
	1	1999-2003	-10.276 (-21.296 to -3.929)	–	0.001
	2	2003-2020	-2.363 (-3.313 to 1.697)	–	0.093
Nonmetropolitan (Rural)	–	1999-2020	–	-2.309 (-3.480 to -1.114)	<0.001

APC, Annual Percent Change; AAPC, Average Annual Percent Change; CI, Confidence Interval. Trend segments indicate periods with significantly different slopes identified by joinpoint analysis.

### 3.2 Gender difference analysis

**Figure 2:**
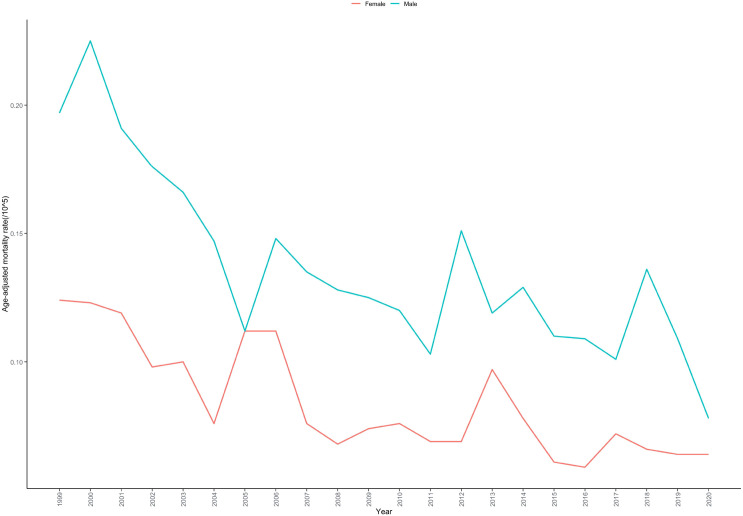
Age-adjusted mortality rate trends for cardiac, mediastinal, and pleural malignancies by sex in the United States, 1999–2020.

Regarding mortality from cardiac, mediastinal, or pleural malignancies, males accounted for 54.04% (n = 2,931) of deaths, while females represented 45.96% (n = 2,493). The AAMR for cardiac, mediastinal, or pleural malignancy-related deaths was significantly higher in males than in females (males: 0.133 per 100,000; females: 0.076 per 100,000). During the study period, the AAMR in males declined from 0.197 in 1999 to 0.078 in 2020, with an AAPC of -3.233% (95% CI: -4.273 to -2.211; P < 0.001). Similarly, for females, the AAMR decreased from 0.124 in 1999 to 0.064 in 2020, with an AAPC of -3.133% (95% CI: -4.296 to -1.980; P < 0.001) (**Tables 1**; **Supplementary Table S2**). Throughout the 1999-2020 period, mortality trends for cardiac, mediastinal, or pleural malignancies showed no joinpoints for either males or females (**Table 2**; **Figure 2**).

### 3.3 Age group difference analysis

**Figure 3:**
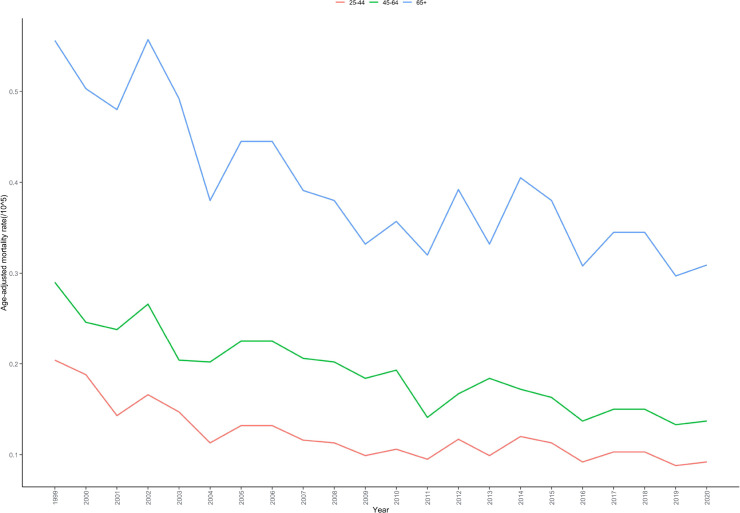
Age-adjusted mortality rate trends for cardiac, mediastinal, and pleural malignancies by age groups in the United States, 1999–2020.

The age distribution of mortality rates underwent significant changes during this period. From 1999-2020, the highest AAMR occurred in the 65+ years age group (0.380 per 100,000; 95% CI: 0.367-0.393), followed by the 45-64 years age group (0.080 per 100,000; 95% CI: 0.076-0.084), while the 25-44 years age group had the lowest AAMR (0.029 per 100,000; 95% CI: 0.027-0.032) (**Table 1**; **Supplementary Table S3**). The 65+ years age group demonstrated a consistent declining trend throughout the study period, with an AAPC of -2.503% (95% CI: -3.377 to -1.627; P < 0.001). The 25-44 years age group showed an overall declining trend with a distinct joinpoint, exhibiting an AAPC of -3.586% (95% CI: -4.519 to -2.258; P < 0.001), characterized by a rapid decline from 1999-2004 (APC = -9.226%; 95% CI: -20.644 to -3.313; P < 0.001) followed by a gradual decline from 2004-2020 (APC = -1.753%; 95% CI: -3.100 to 4.573; P = 0.288). The 45-64 years age group maintained a consistent declining trend with an AAPC of -3.200% (95% CI: -3.820 to -2.566; P < 0.001) (**Table 2**; **Figure 3**).

### 3.4 Racial/ethnic difference analysis

During the study period, White individuals comprised the majority of deaths related to cardiac, mediastinal, or pleural malignancies, accounting for 86.87%, followed by Black or African American individuals at 10.03%. Hispanic or Latino individuals represented 3.10% of the deaths. Regarding AAMR, the White population had the highest rate at 0.109 per 100,000, followed by Black or African American individuals at 0.097 per 100,000, and Hispanic or Latino individuals at 0.059 per 100,000 (**Table 1**; **Supplementary Table S4**).

**Figure 4:**
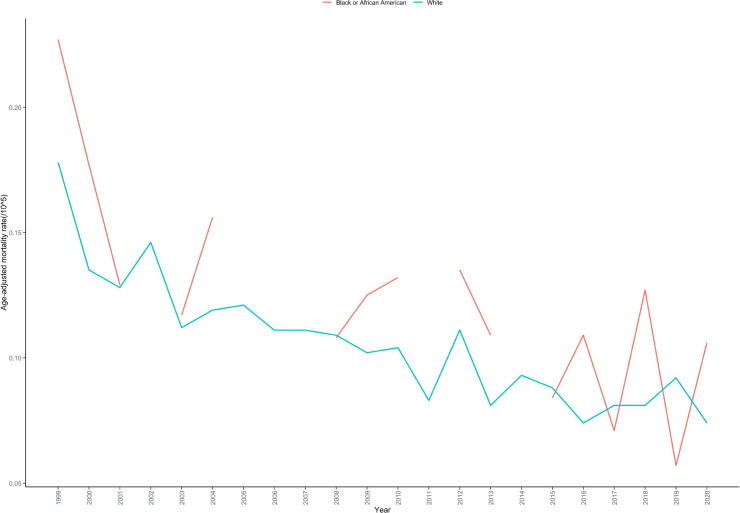
Age-adjusted mortality rate trends for cardiac, mediastinal, and pleural malignancies by race/ethnicity in the United States, 1999–2020.

Due to the low mortality rates and death counts associated with cardiac, mediastinal, or pleural malignancies among Black or African American and Hispanic or Latino populations, with some years recording no deaths, we were unable to conduct overall trend analysis or joinpoint analysis for these groups. In contrast, the White population demonstrated a consistent declining trend throughout the entire study period without joinpoints, with an AAPC of -3.153% (95% CI: -3.808 to -2.471; P < 0.001) (**Table 2**; **Figure 4**).

### 3.5 Geographical region difference analysis

Significant regional disparities existed in mortality rates from cardiac, mediastinal, or pleural malignancies. The West region had the highest AAMR at 0.131 per 100,000 (95% CI: 0.123-0.138), followed by the South at 0.109 per 100,000 (95% CI: 0.103-0.114), the Northeast at 0.097 per 100,000 (95% CI: 0.090-0.104), and the Midwest at 0.085 per 100,000 (95% CI: 0.080-0.091) (**Table 1**; **Supplementary Table S5**). These disparities may reflect differences in population comorbidity profiles, healthcare infrastructure, or regional diagnostic practices.

**Figure 5:**
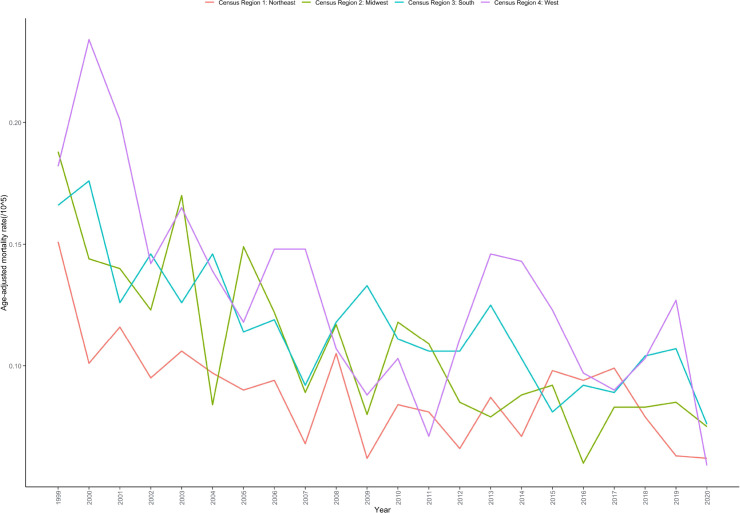
Age-adjusted mortality rate trends for cardiac, mediastinal, and pleural malignancies by geographic region in the United States, 1999–2020.

Despite variations in absolute rates, all four regions experienced significant and sustained declines. The Midwest region demonstrated the greatest decline (AAPC: -3.769%; 95% CI: -4.967 to -2.591; P < 0.001), followed by the West region (AAPC: -3.517%; 95% CI: -5.331 to -1.642; P = 0.001) and the South (AAPC: -2.778%; 95% CI: -3.877 to -1.623; P < 0.001). The Northeast region showed the smallest but still significant decline (AAPC: -2.310%; 95% CI: -3.606 to -1.011; P = 0.001) (**Table 2**; **Figure 5**).

### 3.6 Urban and rural differences

In metropolitan areas, the AAMR for deaths related to cardiac, mediastinal, or pleural malignancies declined from 0.169 per 100,000 in 1999 to 0.064 per 100,000 in 2020. In contrast, in non-metropolitan areas, the AAMR decreased from 0.187 per 100,000 in 1999 to 0.122 per 100,000 in 2020 (**Table 1**; **Supplementary Table S6**).

**Figure 6:**
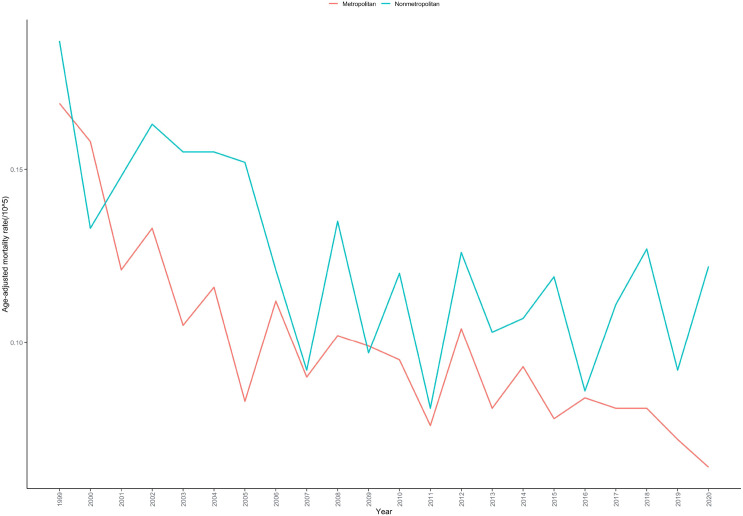
Age-adjusted mortality rate trends for cardiac, mediastinal, and pleural malignancies by urbanization level in the United States, 1999–2020.

In metropolitan areas, a significant declining trend was observed from 1999 to 2003, with an APC of -10.276% (95% CI: -21.296 to -3.929; P = 0.001), followed by a gradual decline from 2003 to 2020 with an APC of -2.363% (95% CI: -3.313 to 1.697; P = 0.093). The overall AAPC for metropolitan areas was -3.922% (95% CI: -4.731 to -2.760; P < 0.001). In contrast, non-metropolitan areas demonstrated a significant and sustained declining trend throughout the 1999-2020 period, with an AAPC of -2.309% (95% CI: -3.480 to -1.114; P < 0.001) (**Table 2**; **Figure 6**).

### 3.7 Trends stratified by state

**Figure 7:**
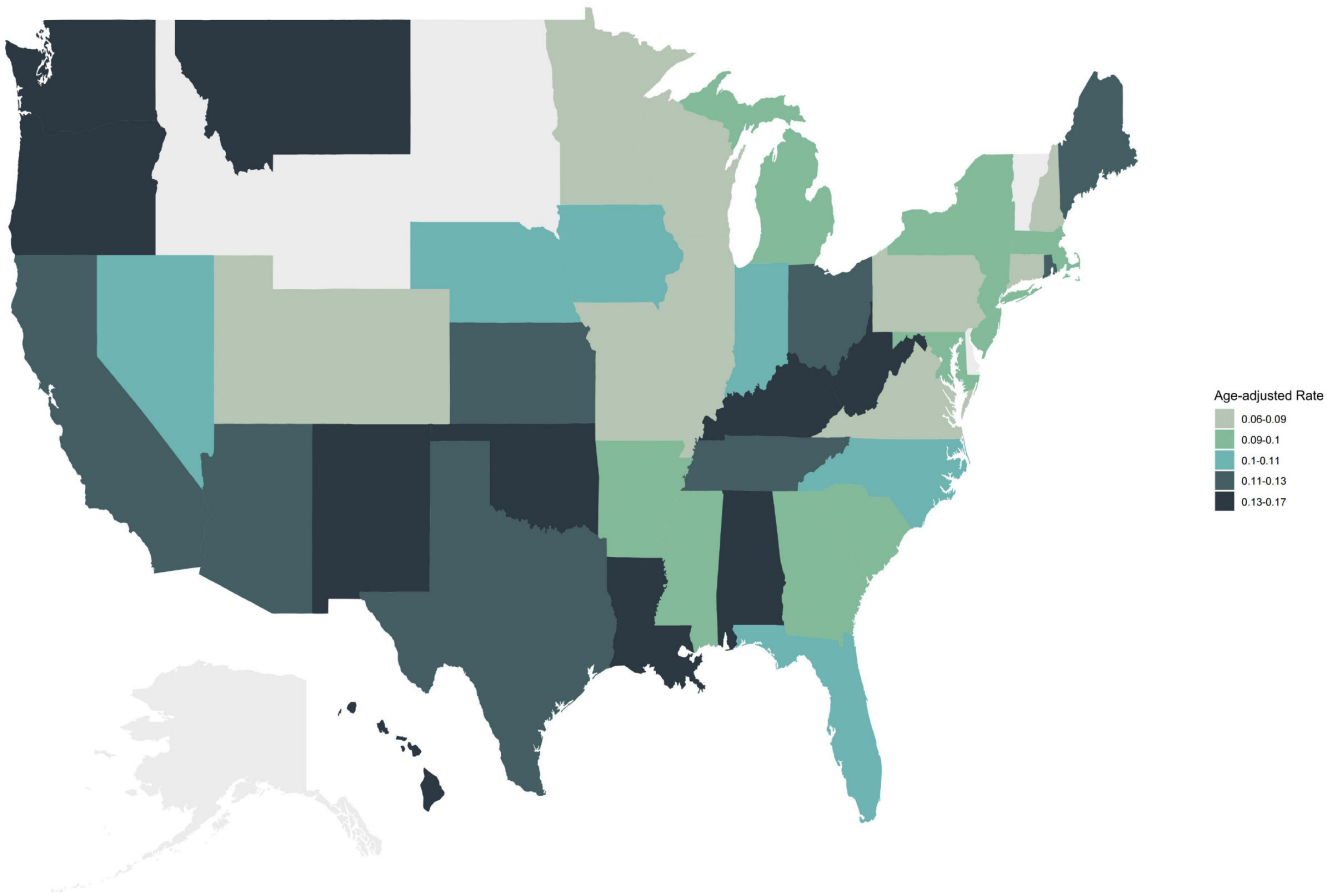
State-level distribution of age-adjusted mortality rates for cardiac, mediastinal, and pleural malignancies in the United States, 1999–2020.

State-level analysis revealed substantial heterogeneity. Washington had the highest AAMR at 0.172 per 100,000 (95% CI: 0.145-0.199), followed by New Mexico with an AAMR of 0.165 per 100,000 (95% CI: 0.115-0.229), West Virginia (0.161 per 100,000; 95% CI: 0.121-0.211), Oklahoma (0.159 per 100,000; 95% CI: 0.126-0.199), and Hawaii (0.158 per 100,000; 95% CI: 0.104-0.229). In contrast, Connecticut had the lowest AAMR at 0.056 per 100,000 (95% CI: 0.038-0.079), followed by Missouri (0.063 per 100,000; 95% CI: 0.049-0.081) and Pennsylvania (0.066 per 100,000; 95% CI: 0.055-0.076). Due to the small number of deaths related to cardiac, mediastinal, or pleural malignancies in Delaware, Idaho, North Dakota, South Dakota, Vermont, and Wyoming, the CDC WONDER database did not calculate AAMRs for these states (**Supplementary Table S7**; **Figure 7**).

## 4 Discussion

This study conducted a comprehensive analysis of mortality rates from cardiac, mediastinal, and pleural malignancies in the United States from 1999-2020, revealing a significant overall declining trend with an AAPC of -4.325%. This decline is closely associated with recent advances in diagnostic and therapeutic technologies for thoracic malignancies (22). Notably, the mortality decline exhibited a biphasic pattern, with a sharp decrease from 1999-2001 (APC: -15.245%) followed by a more gradual decline. This pattern may reflect the improved early detection rates resulting from rapid advances in imaging diagnostic technologies in the late 20th and early 21st centuries (23), as well as the widespread implementation of multidisciplinary treatment approaches in major medical centers (24).

Gender disparities represent one of the key findings of this study. The male AAMR (0.133 per 100,000) was significantly higher than that of females (0.076 per 100,000), consistent with previous research (25). This difference may stem from multiple factors: first, males are more likely to be exposed to carcinogens such as asbestos in occupational environments, increasing the risk of malignant mesothelioma (26); second, sex hormones may play regulatory roles in tumor development, with estrogen demonstrating protective effects against certain thoracic tumors (27); third, historically higher smoking rates among males, with smoking being an independent risk factor for various thoracic malignancies (28). Despite absolute differences in mortality rates between sexes, both demonstrated similar declining trends without joinpoints, suggesting that improvement factors have relatively balanced effects on both sexes.

Age distribution characteristics revealed population-specific disease burden patterns. Individuals aged ≥65 years accounted for 65.08% of total deaths with the highest AAMR (0.380 per 100,000), consistent with the age-dependent characteristics of tumor development (29). Elderly patients often present with multiple comorbidities, poor surgical tolerance, and limited treatment options, which may explain their higher mortality rates (30). Interestingly, the 25-44 years age group exhibited a trend inflection point in 2004, transitioning from rapid early decline to gradual decrease. This may relate to younger patients having better access to aggressive treatments and superior responses to novel therapeutic regimens (31). Additionally, the clinical application of targeted therapies and immune checkpoint inhibitors after 2004 may have provided more pronounced benefits for younger patients (32).

Racial disparity analysis revealed the highest AAMR among White individuals (0.109 per 100,000), followed by Black or African American individuals (0.097 per 100,000) and Hispanic or Latino individuals (0.059 per 100,000). This disparity pattern differs from other cancer types and may reflect the combined influences of genetic susceptibility, environmental exposure, and healthcare accessibility (33). The higher mortality rate among White individuals may be associated with historically higher employment rates in certain high-risk industries (such as shipbuilding and construction) within this population (34). The lower mortality rate among Hispanic or Latino individuals may reflect the “healthy immigrant effect,” where immigrant populations tend to be healthier than native-born populations (35). Due to limited case numbers among minority groups, trend analysis was not feasible, highlighting the need for larger sample studies to adequately assess racial disparities.The higher AAMR among White individuals compared to Black or African American individuals most likely reflects the occupational exposure history specific to the tumor types captured under C38, particularly malignant pleural mesothelioma. White workers were historically overrepresented in asbestos-exposed industries such as shipbuilding, insulation manufacturing, and heavy industrial construction during the peak exposure era of the mid-20th century, which is the predominant driver of C38-associated mortality rather than any differential in biological susceptibility or healthcare access. Additionally, differential cause-of-death coding practices across racial groups in administrative mortality data may contribute to underascertainment of C38 deaths among minority populations, artificially deflating AAMR estimates for these groups. Importantly, the lower population-level AAMR among Black or African American individuals should not be interpreted as reflecting superior cancer outcomes or healthcare access in this population; well-established structural barriers to care including disparities in insurance coverage, specialist access, and treatment quality likely result in worse individual-level prognoses among minority patients who do develop these malignancies, an effect that cannot be captured by population-level AAMR estimates derived from administrative data.

Geographic distribution demonstrated distinct regional characteristics, with the West region having the highest AAMR (0.131 per 100,000) and the Midwest the lowest (0.085 per 100,000). These geographic disparities may relate to multiple factors: first, certain western states historically had extensive mining and shipbuilding industries with higher asbestos exposure risks (36); second, differences exist in cancer screening program coverage and healthcare resource distribution across regions (37); third, environmental factors such as air pollution levels and ultraviolet exposure may influence tumor development (38). Notably, while the Midwest region had the lowest baseline mortality rate, it demonstrated the greatest decline (AAPC: -3.769%), potentially reflecting the effectiveness of healthcare quality improvements and preventive measure implementation in this region (39).

Urban-rural disparity analysis revealed issues of unequal healthcare resource allocation. Metropolitan areas demonstrated faster mortality decline (AAPC: -3.922%) and lower absolute mortality rates in 2020 (0.064 per 100,000), while non-metropolitan areas showed slower decline (AAPC: -2.309%) and relatively higher mortality rates in 2020 (0.122 per 100,000). These disparities may stem from: specialist physician shortages in rural areas requiring patients to travel long distances for specialized care (40); lack of advanced diagnostic equipment and treatment technologies in rural hospitals (41); relatively lower health literacy among rural residents with insufficient participation in early screening (42). The rapid decline in metropolitan areas from 1999-2003 may relate to early adoption of new technologies by large urban medical centers during this period.

State-level analysis revealed substantial heterogeneity, with Washington having the highest AAMR (0.172 per 100,000) and Connecticut the lowest (0.056 per 100,000), representing more than a three-fold difference. These disparities may reflect differences in public health policies, health insurance coverage, and environmental regulation enforcement across states (43). Washington’s higher mortality rate may be associated with the state’s historical shipbuilding industry and related asbestos exposure (44). Insufficient case numbers in certain states precluded AAMR calculations, suggesting potential underdiagnosis or inaccurate cause-of-death coding in these regions (45).

The strengths of this study include the use of national death registry data with large sample sizes and extended temporal coverage, providing comprehensive representation of mortality trends in the U.S. population. Joinpoint regression analysis enabled identification of trend inflection points, offering more precise trend descriptions than simple linear regression. However, this study has limitations: first, the CDC WONDER database relies on death certificate accuracy, and rare tumors may experience diagnostic errors or inaccurate coding, with ICD-10 code C38 encompassing multiple tumor types with significant biological heterogeneity. Second, the absence of individual-level clinical information (tumor staging, treatment regimens, comorbidities) and important socioeconomic variables limits causal inference capabilities. Third, the study period spanned ICD coding system transitions, diagnostic technology advances may have introduced detection bias, and the 2020 COVID-19 pandemic may have affected data quality. Fourth, insufficient case numbers in certain states and minority ethnic groups limited the reliability and generalizability of trend analyses. Fifth, this study analyzed mortality rather than incidence rates, precluding differentiation between mortality decline due to reduced incidence versus improved survival, and did not evaluate patient-centered outcomes such as quality of life.Sixth,Our analysis is restricted to deaths coded under ICD-10 C38 as the underlying cause of death. Mediastinal lymphomas, including Hodgkin’s lymphoma and non-Hodgkin’s lymphoma with mediastinal involvement, are classified under separate ICD-10 codes (C81-C86) and were therefore not included in the present study. This represents an important limitation, as mediastinal lymphomas constitute a substantial and clinically distinct component of the mediastinal tumor burden. Their inclusion would likely have modified the observed mortality trends and demographic patterns, particularly given the remarkable survival improvements achieved through modern lymphoma-directed therapies over the past two decades. Future studies should consider a broader ICD-10 code capture strategy to provide a more complete epidemiological characterization of all mediastinal malignancy-related mortality. Seventh Although ICD-10 code C38 encompasses six distinct diagnostic subcodes representing biologically heterogeneous tumor entities, subcode-level trend analysis was not feasible in the present study due to the extremely small annual death counts associated with individual subcodes, which frequently fell below the CDC WONDER suppression threshold of 10 deaths per cell. This precludes reliable age-adjusted mortality rate estimation and joinpoint regression at the subcode level, and represents an important limitation of using administrative mortality data for the analysis of rare tumor subgroups. Future studies utilizing databases with higher case volumes such as linked SEER-Medicare data or multi-national registry consortia should prioritize subcode-specific analyses to delineate the distinct epidemiological trajectories of cardiac, mediastinal, and pleural malignancies individually. Finally, A fundamental limitation of this study is the inherent biological and clinical heterogeneity of tumors classified under ICD-10 code C38. This code aggregates malignancies with substantially different etiologies, molecular profiles, and prognoses. The observed aggregate mortality trends may therefore reflect the composite influence of divergent subtype-specific trajectories, for example, declining mesothelioma mortality may reflect regulatory controls on asbestos use, while trends in mediastinal malignancies may be more strongly driven by advances in immunotherapy for thymoma. Readers should therefore exercise caution in attributing the overall trends to any single tumor entity. Future studies leveraging databases with higher subtype resolution, such as the SEER program, are needed to disentangle subtype-specific mortality patterns and their determinants.

Through analysis of U.S. national death registry data from 1999-2020, this study provides the first systematic description of mortality trends for cardiac, mediastinal, and pleural malignancies. The findings demonstrate significant overall mortality decline, indicating the effectiveness of therapeutic advances and public health measures. However, males, elderly individuals, specific racial groups, and Western region populations bear higher disease burdens, with rural areas showing markedly slower mortality decline compared to urban areas and three-fold state-level mortality differences. These findings emphasize the need to: strengthen screening and surveillance for high-risk populations; improve specialized healthcare accessibility in rural and medically underserved areas; develop targeted public health strategies; establish comprehensive rare tumor registry systems. Future research should integrate multi-source data, conduct health equity and molecular epidemiology studies, and through multidisciplinary collaboration and sustained public health investment, ensure equitable benefits from medical advances for all populations, ultimately reducing the disease burden of these rare tumors.

## Data Availability

The original contributions presented in the study are included in the article/**Supplementary Material**. Further inquiries can be directed to the corresponding author.
