# Supplementary material for: Mortality trends of cardiac, mediastinal and pleural malignancies in the United States, 1999–2020: a population-based analysis of demographic and geographic disparities
Source: Front Oncol. 2026 May 8;16:1796015. doi: 10.3389/fonc.2026.1796015 (PMC13193811; doi:10.3389/fonc.2026.1796015)
Supplement: Supplementary file 1 [file Table1.doc]

**Supplementary Table S1.Annual Death Counts for Cardiac, Mediastinal, and Pleural Malignancies by Sex and Race/Ethnicity in the United States, 1999-2020**

|  | **Overall** | **Female** | **Male** | **Hispanic or Latino** | **Black or African American** | **White** | **Population** |
| --- | --- | --- | --- | --- | --- | --- | --- |
| **1999** | 299 | 141 | 158 | - | 36 | 256 | 180408769 |
| **2000** | 287 | 126 | 161 | - | 31 | 250 | 181984640 |
| **2001** | 277 | 139 | 138 | - | 21 | 247 | 184305128 |
| **2002** | 270 | 125 | 145 | - | 18 | 248 | 186208028 |
| **2003** | 261 | 123 | 138 | - | 24 | 232 | 188090429 |
| **2004** | 245 | 111 | 134 | - | 27 | 211 | 190205384 |
| **2005** | 232 | 120 | 112 | 10 | 17 | 205 | 192551384 |
| **2006** | 243 | 116 | 127 | - | 26 | 208 | 195019359 |
| **2007** | 210 | 105 | 105 | - | 14 | 190 | 197403777 |
| **2008** | 227 | 100 | 127 | - | 21 | 198 | 199795090 |
| **2009** | 214 | 99 | 115 | - | 25 | 185 | 202107016 |
| **2010** | 224 | 106 | 118 | - | 30 | 189 | 203891983 |
| **2011** | 209 | 97 | 112 | - | 19 | 183 | 206592936 |
| **2012** | 247 | 101 | 146 | - | 27 | 211 | 208826037 |
| **2013** | 240 | 113 | 127 | 12 | 29 | 199 | 211085314 |
| **2014** | 271 | 115 | 156 | 10 | 18 | 243 | 213809280 |
| **2015** | 254 | 107 | 147 | - | 27 | 221 | 216553817 |
| **2016** | 229 | 94 | 135 | - | 29 | 192 | 218641417 |
| **2017** | 259 | 129 | 130 | - | 22 | 228 | 221447331 |
| **2018** | 241 | 111 | 130 | - | 33 | 200 | 223311190 |
| **2019** | 241 | 107 | 134 | 10 | 22 | 209 | 224981167 |
| **2020** | 244 | 108 | 136 | - | 28 | 207 | 226635013 |
| **Total** | 5424 | 2493 | 2931 | 168 | 544 | 4712 | 4473854489 |

**Supplementary Table S2.Annual Age-Adjusted Mortality Rates (per 100,000) for Cardiac, Mediastinal, and Pleural Malignancies by Sex in the United States, 1999-2020**

| **Year** | **Overall** | **Female** | **Male** |
| --- | --- | --- | --- |
| **1999** | 0.182(0.160-0.203) | 0.124(0.102-0.146) | 0.197(0.165-0.230) |
| **2000** | 0.158(0.138-0.177) | 0.123(0.100-0.146) | 0.225(0.190-0.261) |
| **2001** | 0.128(0.112-0.144) | 0.119(0.098-0.140) | 0.191(0.158-0.224) |
| **2002** | 0.143(0.125-0.162) | 0.098(0.079-0.117) | 0.176(0.145-0.206) |
| **2003** | 0.11(0.095-0.125) | 0.1(0.081-0.120) | 0.166(0.136-0.195) |
| **2004** | 0.109(0.094-0.123) | 0.076(0.060-0.092) | 0.147(0.120-0.175) |
| **2005** | 0.121(0.104-0.138) | 0.112(0.090-0.133) | 0.112(0.088-0.136) |
| **2006** | 0.121(0.105-0.137) | 0.112(0.091-0.133) | 0.148(0.120-0.175) |
| **2007** | 0.111(0.095-0.127) | 0.076(0.060-0.092) | 0.135(0.108-0.163) |
| **2008** | 0.109(0.093-0.124) | 0.068(0.053-0.083) | 0.128(0.104-0.152) |
| **2009** | 0.099(0.085-0.114) | 0.074(0.058-0.092) | 0.125(0.101-0.149) |
| **2010** | 0.104(0.089-0.119) | 0.076(0.060-0.093) | 0.12(0.096-0.145) |
| **2011** | 0.076(0.065-0.087) | 0.069(0.054-0.087) | 0.103(0.081-0.124) |
| **2012** | 0.09(0.077-0.103) | 0.069(0.055-0.083) | 0.151(0.125-0.177) |
| **2013** | 0.099(0.085-0.113) | 0.097(0.077-0.117) | 0.119(0.096-0.142) |
| **2014** | 0.093(0.080-0.105) | 0.078(0.063-0.094) | 0.129(0.106-0.151) |
| **2015** | 0.088(0.076-0.100) | 0.061(0.049-0.074) | 0.11(0.090-0.130) |
| **2016** | 0.074(0.063-0.084) | 0.059(0.046-0.073) | 0.109(0.088-0.130) |
| **2017** | 0.081(0.070-0.092) | 0.072(0.058-0.085) | 0.101(0.082-0.120) |
| **2018** | 0.081(0.070-0.092) | 0.066(0.052-0.080) | 0.136(0.111-0.162) |
| **2019** | 0.072(0.061-0.082) | 0.064(0.051-0.078) | 0.109(0.088-0.130) |
| **2020** | 0.074(0.064-0.084) | 0.064(0.051-0.076) | 0.078(0.063-0.094) |
| **Total** | 0.109(0.105-0.112) | 0.076(0.073-0.079) | 0.133(0.128-0.138) |

**Age-Adjusted Rate (95% CI)**

**Supplementary Table S3.Annual Age-Adjusted Mortality Rates (per 100,000) for Cardiac, Mediastinal, and Pleural Malignancies by Age in the United States, 1999-2020**

| **Year** | **25-44** | **45-64** | **65+** |
| --- | --- | --- | --- |
| **1999** | 0.043(0.031-0.060) | 0.123(0.096-0.154) | 0.556(0.476-0.635) |
| **2000** | 0.04(0.028-0.056) | 0.108(0.084-0.137) | 0.503(0.431-0.576) |
| **2001** | 0.041(0.029-0.058) | 0.109(0.085-0.137) | 0.48(0.408-0.552) |
| **2002** | 0.03(0.019-0.044) | 0.088(0.067-0.114) | 0.557(0.477-0.637) |
| **2003** | 0.037(0.025-0.053) | 0.081(0.061-0.106) | 0.492(0.419-0.566) |
| **2004** | 0.03(0.019-0.044) | 0.093(0.072-0.118) | 0.38(0.320-0.440) |
| **2005** | 0.024(0.015-0.037) | 0.077(0.058-0.099) | 0.445(0.375-0.514) |
| **2006** | 0.027(0.017-0.040) | 0.084(0.064-0.107) | 0.445(0.375-0.514) |
| **2007** | 0.025(0.016-0.039) | 0.066(0.049-0.087) | 0.391(0.326-0.456) |
| **2008** | 0.027(0.017-0.040) | 0.07(0.053-0.091) | 0.38(0.319-0.441) |
| **2009** | - | 0.085(0.066-0.107) | 0.332(0.275-0.390) |
| **2010** | 0.039(0.027-0.055) | 0.07(0.053-0.091) | 0.357(0.296-0.417) |
| **2011** | - | 0.062(0.046-0.081) | 0.32(0.267-0.373) |
| **2012** | 0.027(0.017-0.040) | 0.078(0.061-0.100) | 0.392(0.331-0.454) |
| **2013** | 0.028(0.018-0.041) | 0.077(0.059-0.098) | 0.332(0.278-0.386) |
| **2014** | 0.025(0.015-0.038) | 0.075(0.058-0.096) | 0.405(0.346-0.463) |
| **2015** | - | 0.077(0.060-0.099) | 0.38(0.322-0.438) |
| **2016** | 0.025(0.015-0.038) | 0.076(0.059-0.097) | 0.308(0.257-0.358) |
| **2017** | 0.024(0.015-0.037) | 0.072(0.055-0.093) | 0.345(0.293-0.396) |
| **2018** | 0.026(0.017-0.040) | 0.068(0.051-0.088) | 0.345(0.291-0.398) |
| **2019** | 0.029(0.018-0.042) | 0.072(0.055-0.0963) | 0.297(0.250-0.344) |
| **2020** | 0.028(0.018-0.042) | 0.075(0.057-0.096) | 0.309(0.261-0.357) |
| **Total** | 0.029(0.027-0.032) | 0.08(0.076-0.084) | 0.38(0.367-0.393) |

**Age-Adjusted Rate (95% CI)**

**Supplementary Table S4.Annual Age-Adjusted Mortality Rates (per 100,000) for Cardiac, Mediastinal, and Pleural Malignancies by Race in the United States, 1999-2020**

| **Year** | **Hispanic or Latino** | **Black or African American** | **White** |
| --- | --- | --- | --- |
| **1999** | **-** | 0.227(0.157-0.319) | 0.178(0.155-0.201) |
| **2000** | - | 0.177(0.118-0.254) | 0.135(0.117-0.153) |
| **2001** | - | 0.129(0.079-0.199) | 0.128(0.111-0.145) |
| **2002** | - | - | 0.146(0.126-0.165) |
| **2003** | - | 0.117(0.073-0.179) | 0.112(0.096-0.128) |
| **2004** | - | 0.156(0.100-0.233) | 0.119(0.101-0.136) |
| **2005** | - | - | 0.121(0.103-0.139) |
| **2006** | - | 0.128(0.081-0.191) | 0.111(0.095-0.127) |
| **2007** | - | - | 0.111(0.094-0.128) |
| **2008** | - | 0.108(0.064-0.171) | 0.109(0.092-0.125) |
| **2009** | - | 0.125(0.079-0.188) | 0.102(0.086-0.118) |
| **2010** | - | 0.132(0.086-0.194) | 0.104(0.088-0.121) |
| **2011** | - | - | 0.083(0.070-0.096) |
| **2012** | - | 0.135(0.084-0.204) | 0.111(0.094-0.128) |
| **2013** | - | 0.109(0.070-0.162) | 0.081(0.068-0.093) |
| **2014** | - | - | 0.093(0.080-0.105) |
| **2015** | - | 0.084(0.052-0.129) | 0.088(0.075-0.100) |
| **2016** | - | 0.109(0.069-0.164) | 0.074(0.062-0.085) |
| **2017** | - | 0.071(0.041-0.116) | 0.081(0.069-0.092) |
| **2018** | - | 0.127(0.084-0.184) | 0.081(0.069-0.093) |
| **2019** | - | 0.057(0.033-0.092) | 0.092(0.078-0.107) |
| **2020** | - | 0.106(0.067-0.161) | 0.074(0.063-0.085) |
| **Total** | - | 0.097(0.088-0.106) | 0.109(0.105-0.112) |

**Age-Adjusted Rate (95% CI)**

**Supplementary Table S5.Annual Age-Adjusted Mortality Rates (per 100,000) for Cardiac, Mediastinal, and Pleural Malignancies by Urbanization Status in the United States, 1999-2020**

| **Year** | **Metropolitan** | **Non-Metropolitan** |
| --- | --- | --- |
| **1999** | 0.169(0.146-0.192) | 0.187(0.142-0.243) |
| **2000** | 0.158(0.137-0.179) | 0.133(0.096-0.178) |
| **2001** | 0.121(0.104-0.138) | 0.148(0.106-0.199) |
| **2002** | 0.133(0.114-0.152) | 0.163(0.121-0.214) |
| **2003** | 0.105(0.088-0.122) | 0.155(0.116-0.202) |
| **2004** | 0.116(0.098-0.133) | 0.155(0.112-0.209) |
| **2005** | 0.083(0.070-0.097) | 0.152(0.114-0.198) |
| **2006** | 0.112(0.095-0.129) | 0.121(0.088-0.163) |
| **2007** | 0.09(0.075-0.105) | 0.092(0.063-0.131) |
| **2008** | 0.102(0.086-0.118) | 0.135(0.097-0.183) |
| **2009** | 0.099(0.083-0.115) | 0.097(0.063-0.143) |
| **2010** | 0.095(0.079-0.111) | 0.12(0.088-0.161) |
| **2011** | 0.076(0.063-0.089) | 0.081(0.056-0.113) |
| **2012** | 0.104(0.088-0.120) | 0.126(0.084-0.182) |
| **2013** | 0.081(0.068-0.093) | 0.103(0.071-0.143) |
| **2014** | 0.093(0.079-0.106) | 0.107(0.079-0.143) |
| **2015** | 0.078(0.066-0.090) | 0.119(0.087-0.157) |
| **2016** | 0.084(0.070-0.098) | 0.086(0.061-0.118) |
| **2017** | 0.081(0.069-0.093) | 0.111(0.082-0.148) |
| **2018** | 0.081(0.068-0.093) | 0.127(0.091-0.174) |
| **2019** | 0.072(0.061-0.082) | 0.092(0.062-0.131) |
| **2020** | 0.064(0.054-0.074) | 0.122(0.084-0.170) |
| **Total** | 0.109(0.105-0.112) | 0.119(0.111-0.127) |

**Age-Adjusted Rate (95% CI)**

**Supplementary Table S6.Annual Age-Adjusted Mortality Rates (per 100,000) for Cardiac, Mediastinal, and Pleural Malignancies by Census Region in the United States, 1999-2020**

| **Census Region** | **Year** | **Age-Adjusted Rate with 95% CI** |
| --- | --- | --- |
| **Census Region 1: Northeast** | 1999 | 0.151(0.111-0.200) |
| **Census Region 1: Northeast** | 2000 | 0.101(0.070-0.142) |
| **Census Region 1: Northeast** | 2001 | 0.116(0.083-0.159) |
| **Census Region 1: Northeast** | 2002 | 0.095(0.065-0.133) |
| **Census Region 1: Northeast** | 2003 | 0.106(0.074-0.147) |
| **Census Region 1: Northeast** | 2004 | 0.097(0.066-0.138) |
| **Census Region 1: Northeast** | 2005 | 0.09(0.062-0.127) |
| **Census Region 1: Northeast** | 2006 | 0.094(0.065-0.133) |
| **Census Region 1: Northeast** | 2007 | 0.068(0.043-0.103) |
| **Census Region 1: Northeast** | 2008 | 0.105(0.073-0.145) |
| **Census Region 1: Northeast** | 2009 | 0.062(0.039-0.094) |
| **Census Region 1: Northeast** | 2010 | 0.084(0.054-0.125) |
| **Census Region 1: Northeast** | 2011 | 0.081(0.054-0.117) |
| **Census Region 1: Northeast** | 2012 | 0.066(0.045-0.094) |
| **Census Region 1: Northeast** | 2013 | 0.087(0.059-0.123) |
| **Census Region 1: Northeast** | 2014 | 0.071(0.048-0.100) |
| **Census Region 1: Northeast** | 2015 | 0.098(0.069-0.137) |
| **Census Region 1: Northeast** | 2016 | 0.094(0.065-0.133) |
| **Census Region 1: Northeast** | 2017 | 0.099(0.068-0.139) |
| **Census Region 1: Northeast** | 2018 | 0.079(0.050-0.119) |
| **Census Region 1: Northeast** | 2019 | 0.063(0.041-0.093) |
| **Census Region 1: Northeast** | 2020 | 0.062(0.041-0.090) |
| **Census Region 1: Northeast** | Total | 0.097(0.090-0.104) |
| **Census Region 2: Midwest** | 1999 | 0.188(0.146-0.240) |
| **Census Region 2: Midwest** | 2000 | 0.144(0.109-0.187) |
| **Census Region 2: Midwest** | 2001 | 0.14(0.104-0.184) |
| **Census Region 2: Midwest** | 2002 | 0.123(0.092-0.161) |
| **Census Region 2: Midwest** | 2003 | 0.17(0.131-0.216) |
| **Census Region 2: Midwest** | 2004 | 0.084(0.058-0.117) |
| **Census Region 2: Midwest** | 2005 | 0.149(0.112-0.194) |
| **Census Region 2: Midwest** | 2006 | 0.122(0.089-0.164) |
| **Census Region 2: Midwest** | 2007 | 0.089(0.065-0.119) |
| **Census Region 2: Midwest** | 2008 | 0.117(0.086-0.157) |
| **Census Region 2: Midwest** | 2009 | 0.08(0.057-0.109) |
| **Census Region 2: Midwest** | 2010 | 0.118(0.085-0.160) |
| **Census Region 2: Midwest** | 2011 | 0.109(0.079-0.146) |
| **Census Region 2: Midwest** | 2012 | 0.085(0.062-0.115) |
| **Census Region 2: Midwest** | 2013 | 0.079(0.056-0.109) |
| **Census Region 2: Midwest** | 2014 | 0.088(0.064-0.118) |
| **Census Region 2: Midwest** | 2015 | 0.092(0.068-0.122) |
| **Census Region 2: Midwest** | 2016 | 0.06(0.041-0.084) |
| **Census Region 2: Midwest** | 2017 | 0.083(0.060-0.111) |
| **Census Region 2: Midwest** | 2018 | 0.083(0.060-0.113) |
| **Census Region 2: Midwest** | 2019 | 0.085(0.057-0.121) |
| **Census Region 2: Midwest** | 2020 | 0.075(0.055-0.100) |
| **Census Region 2: Midwest** | Total | 0.085(0.080-0.091) |
| **Census Region 3: South** | 1999 | 0.166(0.132-0.200) |
| **Census Region 3: South** | 2000 | 0.176(0.142-0.216) |
| **Census Region 3: South** | 2001 | 0.126(0.100-0.156) |
| **Census Region 3: South** | 2002 | 0.146(0.116-0.175) |
| **Census Region 3: South** | 2003 | 0.126(0.098-0.158) |
| **Census Region 3: South** | 2004 | 0.146(0.116-0.175) |
| **Census Region 3: South** | 2005 | 0.114(0.098-0.158) |
| **Census Region 3: South** | 2006 | 0.119(0.094-0.148) |
| **Census Region 3: South** | 2007 | 0.092(0.070-0.119) |
| **Census Region 3: South** | 2008 | 0.118(0.093-0.148) |
| **Census Region 3: South** | 2009 | 0.133(0.105-0.165) |
| **Census Region 3: South** | 2010 | 0.111(0.087-0.139) |
| **Census Region 3: South** | 2011 | 0.106(0.082-0.135) |
| **Census Region 3: South** | 2012 | 0.106(0.083-0.130) |
| **Census Region 3: South** | 2013 | 0.125(0.098-0.159) |
| **Census Region 3: South** | 2014 | 0.103(0.081-0.124) |
| **Census Region 3: South** | 2015 | 0.081(0.064-0.101) |
| **Census Region 3: South** | 2016 | 0.092(0.072-0.116) |
| **Census Region 3: South** | 2017 | 0.089(0.070-0.108) |
| **Census Region 3: South** | 2018 | 0.104(0.082-0.130) |
| **Census Region 3: South** | 2019 | 0.107(0.085-0.134) |
| **Census Region 3: South** | 2020 | 0.076(0.060-0.092) |
| **Census Region 3: South** | Total | 0.109(0.103-0.114) |
| **Census Region 4: West** | 1999 | 0.182(0.137-0.236) |
| **Census Region 4: West** | 2000 | 0.234(0.186-0.292) |
| **Census Region 4: West** | 2001 | 0.201(0.158-0.252) |
| **Census Region 4: West** | 2002 | 0.142(0.107-0.186) |
| **Census Region 4: West** | 2003 | 0.165(0.125-0.214) |
| **Census Region 4: West** | 2004 | 0.139(0.104-0.183) |
| **Census Region 4: West** | 2005 | 0.118(0.087-0.156) |
| **Census Region 4: West** | 2006 | 0.148(0.112-0.192) |
| **Census Region 4: West** | 2007 | 0.148(0.112-0.191) |
| **Census Region 4: West** | 2008 | 0.107(0.077-0.146) |
| **Census Region 4: West** | 2009 | 0.088(0.063-0.119) |
| **Census Region 4: West** | 2010 | 0.103(0.075-0.138) |
| **Census Region 4: West** | 2011 | 0.071(0.050-0.098) |
| **Census Region 4: West** | 2012 | 0.111(0.081-0.149) |
| **Census Region 4: West** | 2013 | 0.146(0.113-0.187) |
| **Census Region 4: West** | 2014 | 0.143(0.112-0.182) |
| **Census Region 4: West** | 2015 | 0.123(0.091-0.163) |
| **Census Region 4: West** | 2016 | 0.097(0.073-0.127) |
| **Census Region 4: West** | 2017 | 0.09(0.067-0.118) |
| **Census Region 4: West** | 2018 | 0.103(0.077-0.136) |
| **Census Region 4: West** | 2019 | 0.127(0.097-0.164) |
| **Census Region 4: West** | 2020 | 0.059(0.042-0.081) |
| **Census Region 4: West** | Total | 0.131(0.123-0.138) |

**Supplementary Table S7.Age-Adjusted Mortality Rates (per 100,000) for Cardiac, Mediastinal, and Pleural Malignancies by State in the United States, 1999-2020**

| **State** | **Age-Adjusted Rate with 95% CI** |
| --- | --- |
| **Washington** | 0.172(0.145-0.199) |
| **New Mexico** | 0.165(0.115-0.229) |
| **West Virginia** | 0.161(0.121-0.211) |
| **Oklahoma** | 0.159(0.126-0.199) |
| **Hawaii** | 0.158(0.104-0.229) |
| **Oregon** | 0.157(0.125-0.193) |
| **Montana** | 0.156(0.095-0.240) |
| **Alabama** | 0.146(0.117-0.176) |
| **Louisiana** | 0.131(0.104-0.162) |
| **Kentucky** | 0.13(0.104-0.162) |
| **Texas** | 0.128(0.115-0.142) |
| **Rhode Island** | 0.125(0.077-0.191) |
| **California** | 0.12(0.111-0.130) |
| **Kansas** | 0.119(0.086-0.162) |
| **Maine** | 0.116(0.073-0.174) |
| **Tennessee** | 0.116(0.094-0.138) |
| **Arizona** | 0.113(0.092-0.133) |
| **Ohio** | 0.113(0.096-0.130) |
| **Nevada** | 0.11(0.076-0.155) |
| **Total** | 0.109(0.105-0.112) |
| **Nebraska** | 0.108(0.073-0.153) |
| **Indiana** | 0.105(0.085-0.125) |
| **North Carolina** | 0.104(0.085-0.123) |
| **Florida** | 0.102(0.090-0.113) |
| **Iowa** | 0.102(0.076-0.133) |
| **Michigan** | 0.1(0.085-0.115) |
| **South Carolina** | 0.098(0.075-0.125) |
| **New Jersey** | 0.097(0.079-0.115) |
| **New York** | 0.097(0.085-0.109) |
| **Arkansas** | 0.095(0.068-0.130) |
| **Maryland** | 0.094(0.073-0.119) |
| **Mississippi** | 0.094(0.066-0.130) |
| **Georgia** | 0.091(0.074-0.109) |
| **Massachusetts** | 0.091(0.072-0.114) |
| **New Hampshire** | 0.09(0.053-0.143) |
| **Utah** | 0.086(0.054-0.130) |
| **Colorado** | 0.085(0.064-0.111) |
| **Illinois** | 0.085(0.072-0.099) |
| **Minnesota** | 0.08(0.061-0.104) |
| **Virginia** | 0.078(0.062-0.095) |
| **Wisconsin** | 0.078(0.062-0.098) |
| **Pennsylvania** | 0.066(0.055-0.076) |
| **Missouri** | 0.063(0.049-0.081) |
| **Connecticut** | 0.056(0.038-0.079) |
| **Delaware** | - |
| **Idaho** | - |
| **North Dakota** | - |
| **South Dakota** | - |
| **Vermont** | - |
| **Wyoming** | - |
